# Connexin hemichannel inhibition ameliorates epidermal pathology in a mouse model of keratitis ichthyosis deafness syndrome

**DOI:** 10.1038/s41598-021-03627-8

**Published:** 2021-12-16

**Authors:** Caterina Sellitto, Leping Li, Thomas W. White

**Affiliations:** Department of Physiology and Biophysics, Stony Brook University, T5-147, Basic Science Tower, Stony Brook, NY 11794-8661 USA

**Keywords:** Skin diseases, Biophysics, Permeation and transport, Mechanisms of disease

## Abstract

Mutations in five different genes encoding connexin channels cause eleven clinically defined human skin diseases. Keratitis ichthyosis deafness (KID) syndrome is caused by point mutations in the *GJB2* gene encoding Connexin 26 (Cx26) which result in aberrant activation of connexin hemichannels. KID syndrome has no cure and is associated with bilateral hearing loss, blinding keratitis, palmoplantar keratoderma, ichthyosiform erythroderma and a high incidence of childhood mortality. Here, we have tested whether a topically applied hemichhanel inhibitor (flufenamic acid, FFA) could ameliorate the skin pathology associated with KID syndrome in a transgenic mouse model expressing the lethal Cx26-G45E mutation. We found that FFA blocked the hemichannel activity of Cx26-G45E in vitro, and substantially reduced epidermal pathology in vivo, compared to untreated, or vehicle treated control animals. FFA did not reduce the expression of mutant connexin hemichannel protein, and cessation of FFA treatment allowed disease progression to continue. These results suggested that aberrant hemichannel activity is a major driver of skin disease in KID syndrome, and that the inhibition of mutant hemichannel activity could provide an attractive target to develop novel therapeutic interventions to treat this incurable disease.

## Introduction

Connexin mutations cause human genetic diseases affecting epithelial tissues^1^. For example, mutations in five connexin genes have been linked to eleven genodermatoses, six of which result from mutations in connexin26 (Cx26)^2,3^. Keratitis ichthyosis deafness (KID) syndrome was linked to mutations in Cx26 in 2002^4,5^. Increased activity of Cx26 hemichannels emerged as a common gain of function among the causative mutations^6–12^, and mouse models reproduced the epidermal pathology and displayed hemichannel activity in the affected keratinocytes^13,14^.

Connexins are subunits of gap junction channels, which allow passage of small molecules between adjacent cells^15,16^. Connexins form hemichannels in the ER-Golgi pathway^17,18^, and hemichannels are delivered to the plasma membrane, where they can become active channels, or dock with another hemichannel from an adjacent cell to form a gap junction channel^19^. Hemichannels allow the transmembrane flux of molecules, contributing to a wide range of physiological responses^20,21^.

KID syndrome is a dysregulated hemichannel disorder characterized by deafness, keratitis, palmoplantar keratoderma, and ichthyosiform erythroderma^3,22^. Among KID patients, a genotype–phenotype correlation has emerged^10,23^. Patients with the Cx26-D50N mutation live into adulthood, and frequently develop squamous cell carcinomas^24,25^. In contrast, approximately 10% of KID patients die in early childhood, and two frequent mutations in this cohort, Cx26-G45E or Cx26-A88V, are always lethal^2,22^. For KID syndrome, hemichannel inhibition could have therapeutic value^26^. Flufenamic acid (FFA) is a non-specific connexin inhibitor that blocks hemichannels and gap junction channels^27,28^. FFA has previously been used in humans with side effects limited to gastrointestinal problems when used orally^29–31^. Commercially available topical creams containing FFA are used in humans for pain and inflammation associated with musculoskeletal and joint disorders and provide an ideal route of drug delivery for testing in mouse models of skin disease.

We examined whether topically applied FFA could ameliorate the skin disease associated with KID syndrome in a transgenic mice expressing the Cx26-G45E mutation^13^. We found that FFA blocked the hemichannel activity of Cx26-G45E in vitro, and reduced epidermal pathology in vivo. FFA did not reduce connexin hemichannel expression, and cessation of treatment allowed disease progression.

## Results

### FFA blocked mutant Cx26 hemichannels

**Figure 1:**
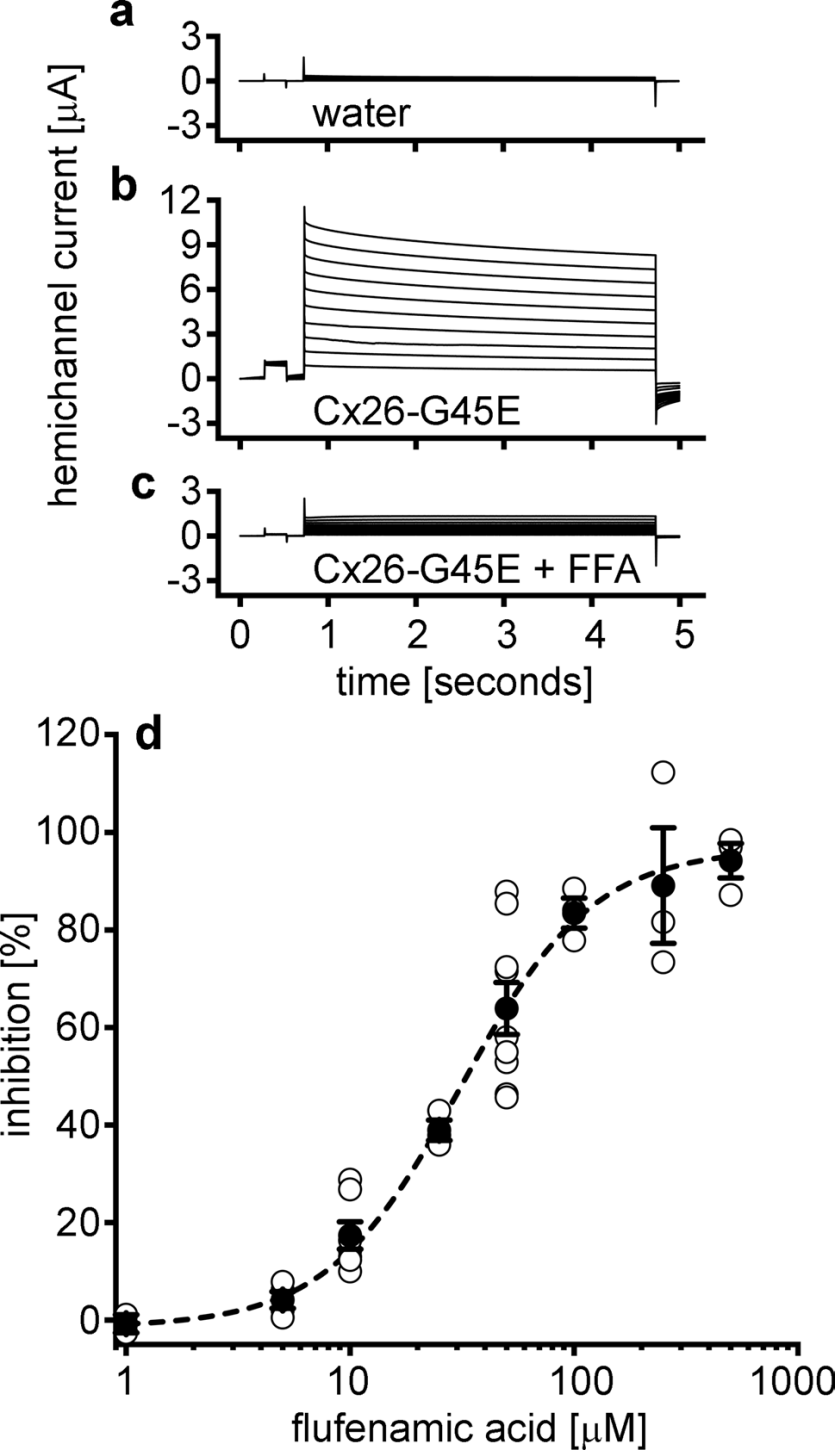
FFA blocked mutant Cx26-G45E hemichannels in vitro. (**a**) Control *Xenopus* oocytes injected with water instead of Cx26-G45E mRNA displayed no membrane hemichannel current. (**b**) Large hemichannel currents were recorded in oocytes injected with Cx26-G45E mRNA. (**c**) Perfusion of these cells with 50 µM FFA substantially inhibited the hemichannel currents. (**d**) Fitting of a dose response curve yielded an IC_50_ ≈ 31 µM for FFA. n = 3–9 oocytes per tested FFA concentration. Filled symbols are means ± SE, open symbols are raw data.

We tested the ability of FFA to block hemichannel activity of the Cx26-G45E mutation in vitro. Control *Xenopus* oocytes injected with water showed negligible membrane current when depolarizing voltage steps were applied (Fig. 1a). As previously reported^6,8^, the Cx26-G45E mutation induced large membrane currents in single oocytes (Fig. 1b). Perfusion of 50 µM FFA blocked these hemichannel currents (Fig. 1c). Fitting of a dose response curve revealed that FFA blocked hemichannel currents induced by Cx26-G45E with an IC_50_ ≈ 31 µM (Fig. 1d). Thus, FFA can inhibit aberrant hemichannel activity associated with Cx26 KID mutations in vitro.

### Quantitative assessment of skin disease in transgenic mice

**Figure 2:**
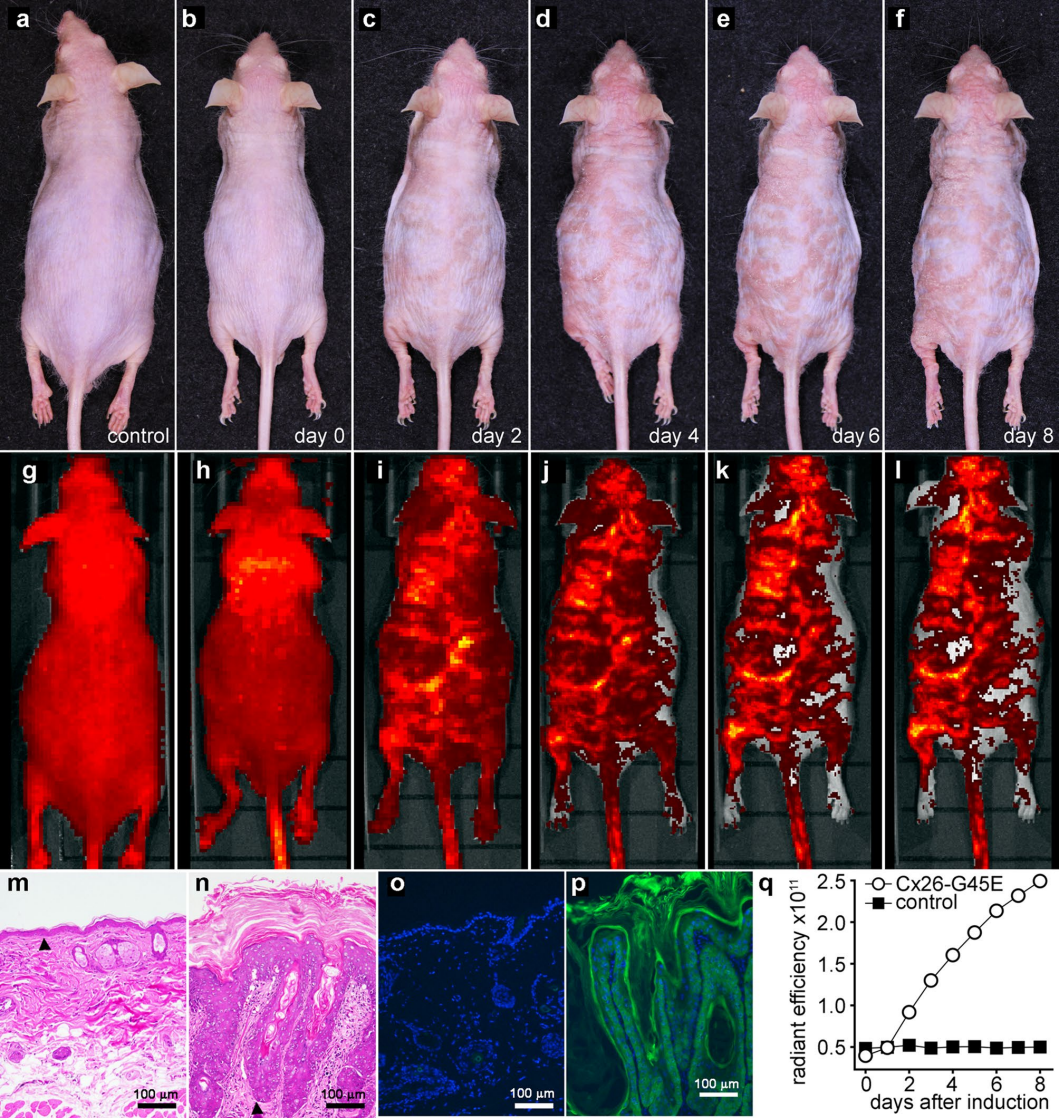
Quantitation of skin disease. (**a**) A wild-type mouse, and (**b**) a Cx26-G45E transgenic mouse had normal skin before doxycycline induction. (**c**–**f**) KID lesions appeared on the transgenic on day 2, and worsened through day 8 after induction. IVIS imaging showed low background fluorescence in the (**g**) wild-type and (**h**) transgenic animals before induction. (**i**–**l**) EGFP fluorescence increased for the transgenic from days 2–8. (**m**) Normal epidermis in the control mouse. (**n**) The transgenic displayed KID pathology. (**o**) No EGFP fluorescence was detected in control skin. (**p**) An EGFP signal was observed in the thickened epidermis of the transgenic. (**q**) Plots of the total fluorescent radiant efficiency against the time after doxycycline induction. Black arrowheads mark the epidermal/dermal boundary.

Mice with inducible expression of the human Cx26-G45E mutation in keratinocytes replicate the epidermal pathology of KID syndrome. They also express enhanced green fluorescent protein (EGFP) in keratinocytes^13,26^, allowing epidermal thickness to be evaluated using an In Vivo Imaging System (IVIS). Figure 2 shows the correlation between EGFP fluorescence and epidermal pathology following the induction of a Cx26-G45E transgenic mouse by doxycycline. Wild-type control and Cx26-G45E transgenic mice had normal skin prior to doxycycline induction (Fig. 2a,b). KID lesions appeared on the transgenic animal by day 2 of induction, and progressively worsened through day 8 (Fig. 2c,f). Whole body fluorescent images showed that prior to induction, the wild-type control and transgenic animals had similar low levels of background fluorescence (Fig. 2g,h). EGFP fluorescent intensity increased for the transgenic animal from days 2–8, and correlated spatially with the visible KID lesions (Fig. 2i–l). Histological sections (Fig. 2m,n) obtained after induction showed normal epidermis in the control mouse, whereas the Cx26-G45E transgenic animal displayed typical features of KID syndrome, including acanthosis, papillomatosis, and a thickened stratum corneum^13^. Frozen sections showed an absence of EGFP fluorescence in control skin (Fig. 2o), and a strong EGFP signal throughout the thickened epidermis of Cx26-G45E transgenic skin (Fig. 2p). Plotting the total fluorescent radiant efficiency against the time after doxycycline induction (Fig. 2q) demonstrated an increase in fluorescence in the transgenic mouse that was temporally correlated with worsening pathology. Therefore, IVIS imaging of EGFP fluorescence provided a spatially and temporally correlated measure of epidermal pathology in Cx26-G45E transgenic mice.

**Figure 3:**
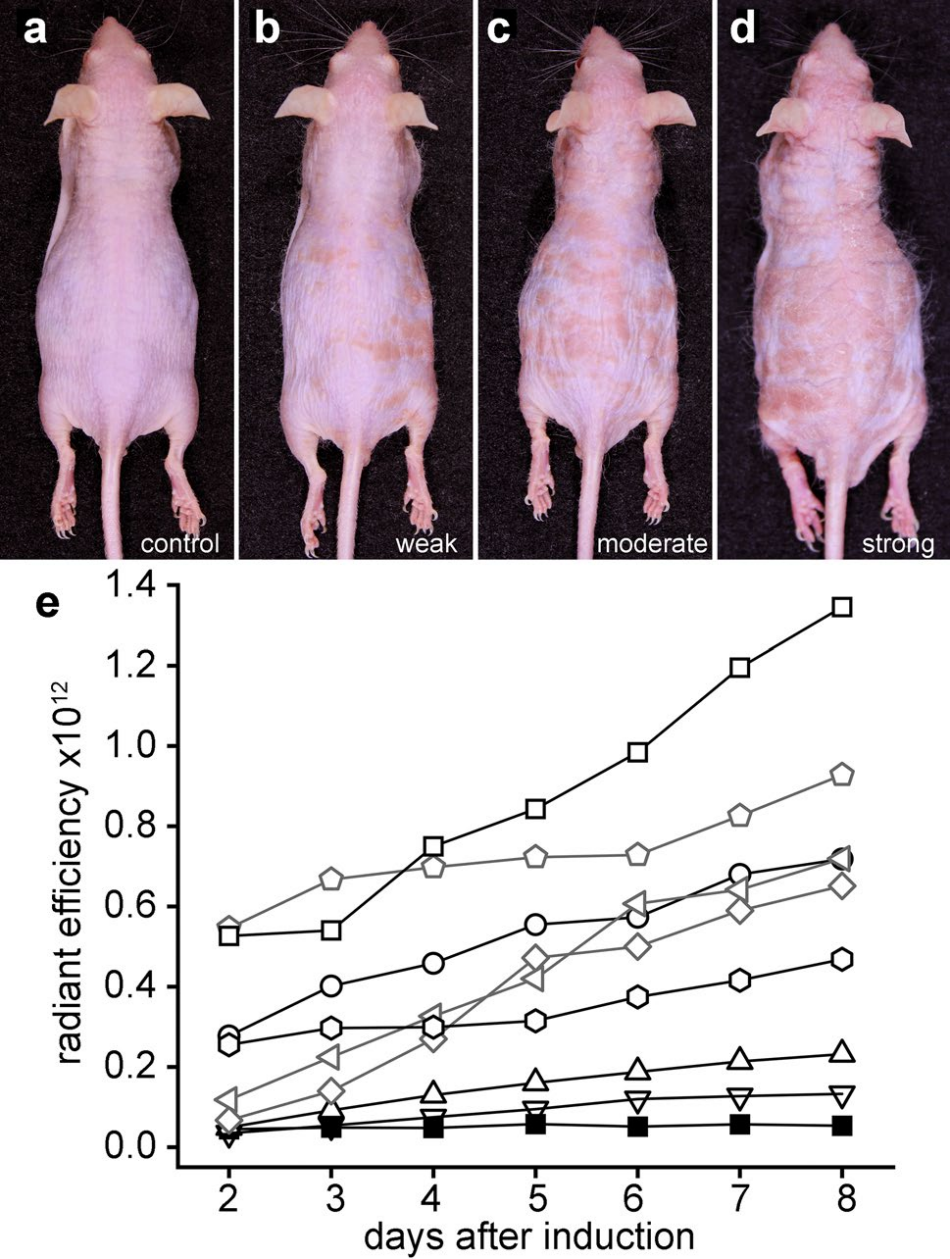
Cx26-G45E transgenic mice showed inter-animal variation in the extent of epidermal pathology. (**a**) Wild-type control mice never displayed KID pathology on a doxycycline containing diet. (**b**–**d**) The epidermal pathology that developed in Cx26-G45E transgenic mice varied from weak to strong. (**e**) Representative EGFP fluorescence data showed that all Cx26-G45E transgenic animals (open symbols) produced a steadily increasing response that was higher than that of the wild-type control (filled squares), but the slope and magnitude of the radiant efficiency varied between individual mice.

Variation in epidermal pathology was observed between individual Cx26-G45E mice, most likely due to their outbred and undefined genetic background^13,32^. Figure 3 shows representative examples of the variability in skin disease development. Wild-type control mice never exhibited erythrokeratoderma while on a doxycycline containing diet (Fig. 3a). All Cx26-G45E mice developed epidermal pathology, but the response varied from weak to strong (Fig. 3b–d). Similar trends were observed in the EGFP fluorescence data, which showed that all animals produced an increasing response higher than controls, but the slope and magnitude of the radiant efficiency varied (Fig. 3e). Despite inter-animal variability, the strong intra-animal correlation between the IVIS measurement of EGFP fluorescence and severity of disease was observed. To ensure that comparisons between untreated, drug, and vehicle treated mice were made between animals exhibiting similar pathology, we adopted the following experimental approach: Groups of transgenic mice were induced with doxycycline and subjected to daily IVIS imaging to select cohorts with similar levels of increase in total fluorescent radiant efficiency and disease progression. These cohorts were then divided equally among the different treatment groups in experiments testing the efficacy of FFA treatment.

### Mobilisin treatment reduced epidermal pathology

**Figure 4:**
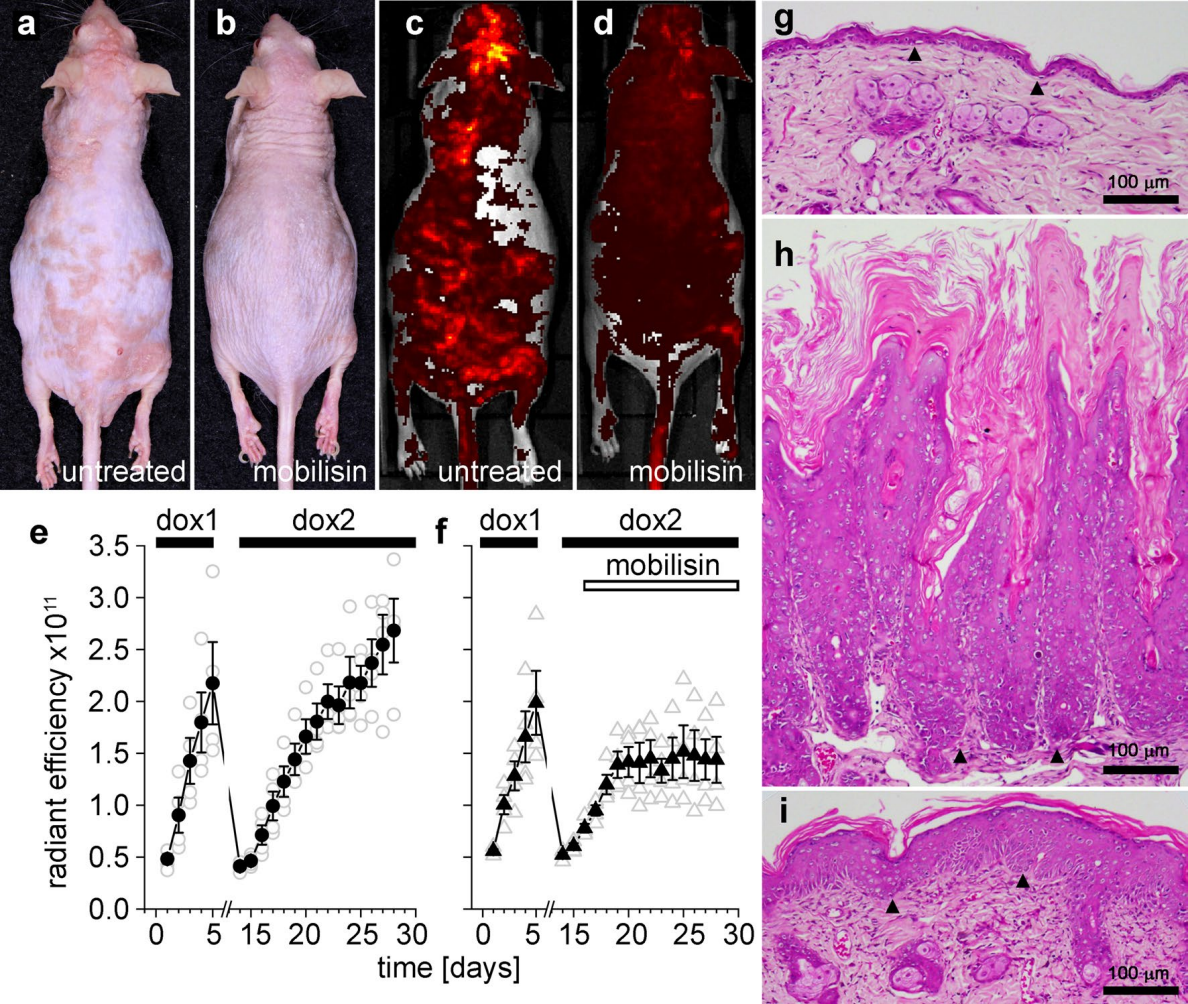
Mobilisin treatment reduced epidermal pathology. Untreated mice (**a**) displayed more severe skin pathology than Mobilisin treated animals (**b**) that correlated well with IVIS images of EGFP fluorescent intensity (**c**,**d**). Radiant efficiency plots for untreated (**e**), or Mobilisin treated (**f**), mice after an initial doxycycline induction (dox1) without FFA treatment, removal of doxycycline, then a second doxycycline induction (dox2) with FFA treatment of one group of animals. Wild-type mice (**g**) showed normal epidermal structure and thickness. Untreated Cx26-G45E transgenic mice (**h**) displayed typical KID pathology. Mobilisin treatment (**i**) significantly reduced, but did not eliminate, hyperkeratosis. Filled symbols are means ± SE, open symbols are raw data. n = 4/group. Black arrowheads mark the epidermal/dermal boundary.

To test if topical treatment with FFA could ameliorate the skin disease associated with KID syndrome, we treated Cx26-G45E transgenic mice with topically applied FFA using commercially available Mobilisin cream (Fig. 4). Transgenic animals were induced with doxycycline containing chow and EGFP fluorescence was quantitatively monitored in the skin using IVIS imaging for 5 days to ensure equal disease progression between the control and Mobilisin treated groups. Animals were then taken off doxycycline and allowed to recover for 7 days, until KID lesions disappeared and EGFP fluorescence returned to baseline. Mice were then induced with doxycycline a second time, with or without twice daily treatment with Mobilisin beginning on the third day of the second induction. Visual observation showed a marked reduction of KID lesion severity in the Mobilisin treated animals compared to the untreated controls. Two weeks after the second doxycycline induction, the untreated Cx26-G45E animals (Fig. 4a) developed obvious skin lesions that were greatly diminished in the animals receiving Mobilisin treatment (Fig. 4b), and these differences in lesions correlated well with the corresponding images of EGFP fluorescence (Fig. 4c,d). These findings were corroborated by the aggregate IVIS imaging data obtained for all eight mice (Fig. 4e,f). During the first induction on days 0 to 5, in the absence of hemichannel inhibition, the radiant efficiency increased in a similar manner for both groups of animals as disease progression worsened (P > 0.05, student’s t-test). Between days 6 to 14, the fluorescence diminished to baseline following withdrawal of doxycycline. On days 19–28, the four animals topically treated with Mobilisin stabilized at a constant fluorescent level after treatment. In contrast, the radiant efficiency in the four untreated mice continued to increase as their skin lesions worsened, and the observed values were 47% higher than those in the Mobilisin treated group (P < 0.05). Skin biopsies were taken on day 28 and processed for histology. The untreated Cx26-G45E animals displayed typical KID pathology, that included extensive hyperkeratosis, osteal plugging, acanthosis, and papillomatosis compared to a negative control wild-type mouse (Fig. 4g,h). The Mobilisin treated animals (Fig. 4i) still exhibited hyperkeratosis, but to a greatly reduced extent compared to the untreated animals. These data showed that a topically applied connexin hemichannel inhibitor significantly reduced epidermal thickening in a mouse model of KID syndrome.

### FFA was required for reduced pathology

**Figure 5:**
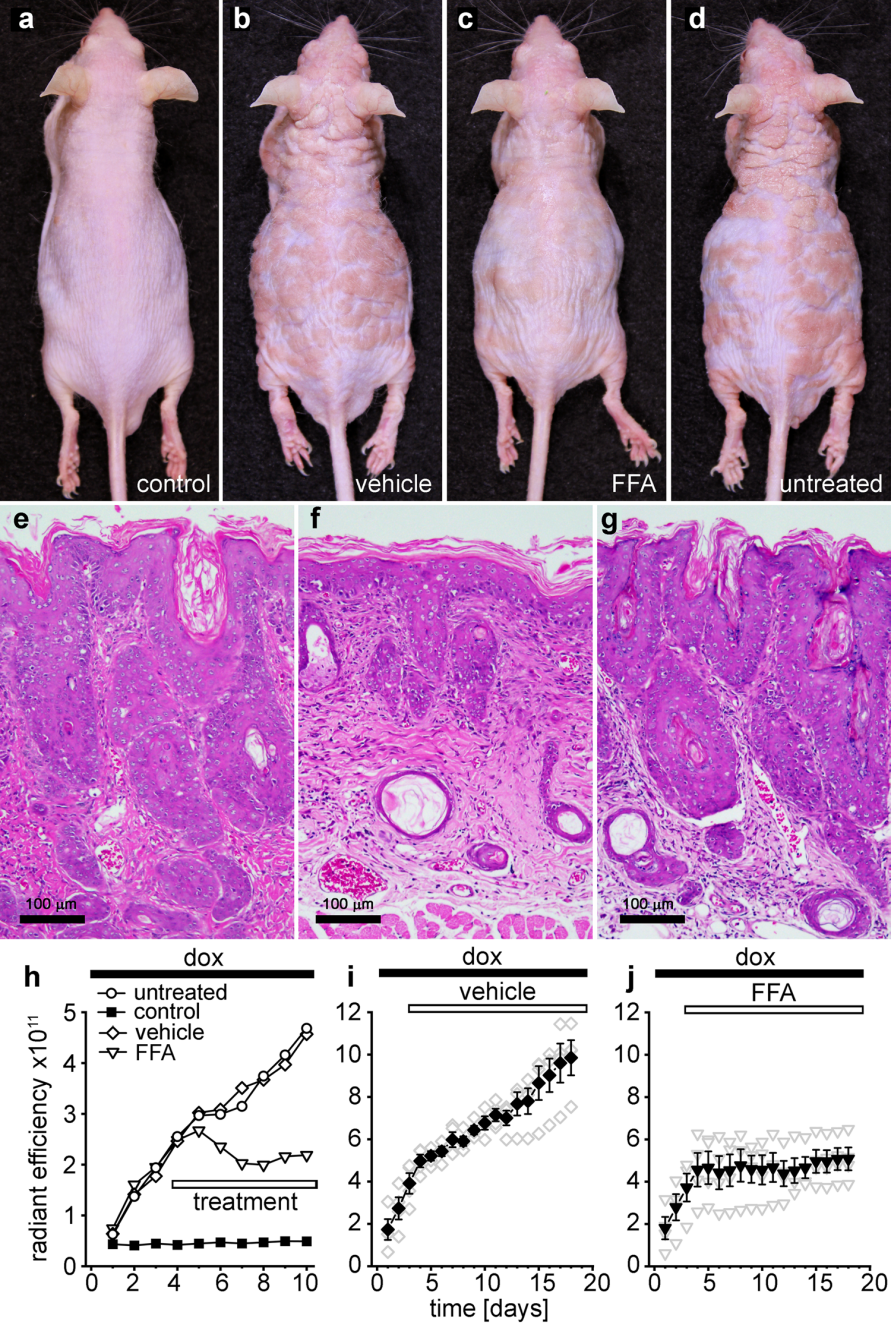
FFA was required for amelioration of KID pathology. (**a**–**d**), Images of a control wild-type mouse (**a**), a vehicle treated transgenic mouse (**b**), a FFA treated transgenic mouse (**c**) and an untreated transgenic mouse (**d**). FFA treatment reduced (**f**) skin pathology seen in untreated (**e**), or vehicle treated (**g**) transgenic mice. Prior to FFA or vehicle treatment, the radiant efficiency (**h**) increased for all three animals. The mouse topically treated with FFA stabilized at a constant fluorescent value. Radiant efficiency in the untreated, or vehicle treated mice increased. Treatment of groups of animals (n = 4) with vehicle (**i**) or topical FFA (**j**) for 16 days produced similar results. Filled symbols are means ± SE, open symbols are raw data.

Emollients are used for palliative relief in KID syndrome^33^. To confirm that non-specific effects of emollient were not responsible for the improved pathology seen in Fig. 4, we generated topical creams that were identical in composition with the exception of the presence or absence of FFA. FFA was dissolved in DMSO, mixed with Eucerin cream (final FFA concentration was 30 mM) and applied once daily. As controls, transgenic animals were either untreated, or treated with an equal volume of DMSO/Eucerin vehicle (~ 0.5 ml/ mouse). Visual observation showed a reduction of KID lesion severity in the FFA treated animal compared to either the untreated, or vehicle controls after 6 days of treatment (Fig. 5a–d). Skin biopsies obtained after 6 days of treatment confirmed that the vehicle control, or untreated Cx26-G45E animals displayed typical KID pathology. Similar to the results in Fig. 4, The FFA/Eucerin treated animal (Fig. 5f) had reduced skin pathology when compared to the untreated (Fig. 5e), or DMSO/Eucerin vehicle control (Fig. 5g) animals. These findings were supported by IVIS imaging data (Fig. 5h). Prior to topical treatment, the radiant efficiency increased in a similar manner for all three Cx26-G45E transgenic animals. Between days 5 to 10, the mouse topically treated with FFA/Eucerin stabilized at a constant fluorescent value. In contrast, the radiant efficiency in the untreated, or DMSO/Eucerin vehicle treated mice continued to increase as their skin lesions worsened. To confirm this result, we treated groups of animals (n = 4) with topical FFA/Eucerin and DMSO/Eucerin vehicle for 16 days (Fig. 5i,j). The mean radiant efficiency of EGFP fluorescence was reduced 50% in FFA/Eucerin treated animals (P < 0.05) after 16 days of topical application. These data showed that the improvement in skin pathology required the presence of the hemichannel inhibitor FFA.

### FFA did not affect mutant connexin expression

**Figure 6:**
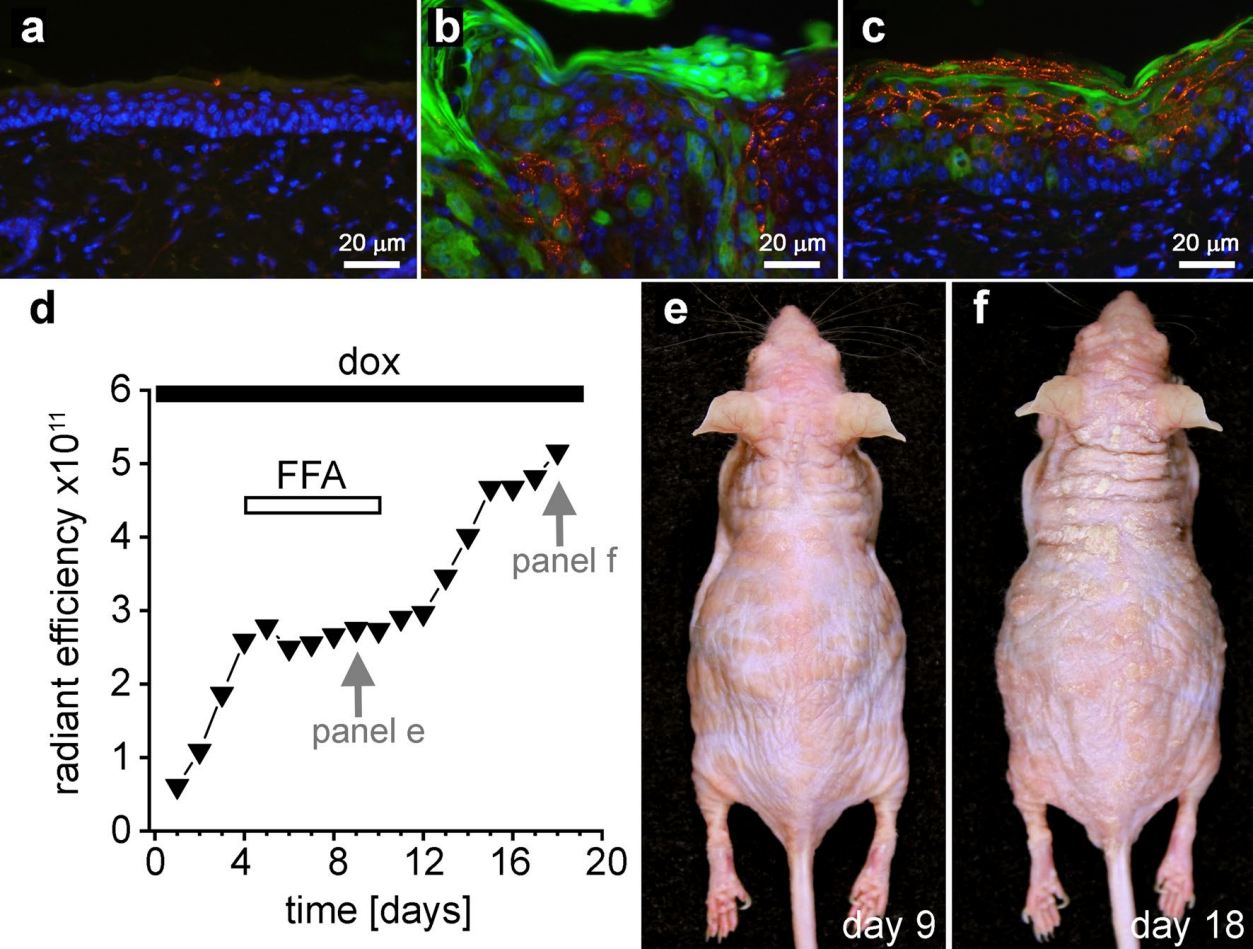
FFA did not inhibit mutant connexin expression and cessation of treatment allowed progression of epidermal pathology. No EGFP or Cx26 signals were detected in wild-type control epidermis (**a**). Untreated (**b**) and FFA treated (**c**) transgenic mice showed an induction of EGFP in keratinocytes and a punctate pattern of Cx26 staining. A transgenic animal was treated for 6 days with FFA, and then treatment ended and the mouse was followed for an additional 8 days. IVIS imaging (**d**) showed that fluorescent efficiency increased prior to FFA treatment, remained stable during treatment, and resumed increasing after treatment was suspended. Images of the mouse taken during (**e**), and following cessation of FFA treatment (**f**) confirmed that FFA withdrawal correlated with increased epidermal pathology.

Development of skin disease in the mouse model required induction of mutant connexin expression by doxycycline. This raised the concern that topical treatment with FFA may have interfered with the transgenic expression of mutant connexins, rather than pharmacologically inhibited their activity. To exclude this possibility, skin biopsies were obtained from wild-type control, untreated, and FFA/Eucerin treated transgenic animals and stained with an antibody against Cx26. Fluorescent microscopy showed no detectable EGFP or Cx26 signals in wild-type control epidermis (Fig. 6a). Both untreated (Fig. 6b) and FFA/Eucerin treated (Fig. 6c) Cx26-G45E transgenic mice showed a strong induction of EGFP in keratinocytes and a punctate pattern of Cx26 staining. These data showed that the improvement in skin pathology following FFA treatment was not due to off-target interference with the transgenic expression of the mutated connexin.

### Cessation of FFA treatment allowed resumption of pathology

If inhibition of aberrant hemichannel activity caused the arrest of skin disease development, then cessation of topical treatment with FFA should allow disease progression to continue. To qualitatively test this, we induced a Cx26-G45E transgenic animal, treated it for 6 days with topical FFA/Eucerin, and then suspended treatment and followed the mouse for 8 additional days. IVIS imaging (Fig. 6d) data revealed that prior to the FFA/Eucerin treatment, EGFP fluorescent efficiency steadily increased. During the FFA/Eucerin treatment, EGFP fluorescence remained at a stable level, and disease progression was arrested. When FFA/Eucerin treatment was terminated, the increase in fluorescent efficiency resumed. Comparison of images of the mouse during, and following cessation of treatment confirmed that the increase in EGFP fluorescence after FFA withdrawal correlated with increased epidermal pathology (Fig. 6e,f). These data suggested that continued topical treatment with FFA was required to sustain the amelioration of skin pathology associated with KID syndrome.

## Discussion

KID syndrome is a rare disorder^34^. Infectious complications, increased cancer risk and respiratory dysfunction contribute to mortality among patients^22,24,35,36^. Current treatments for skin pathology include antibiotic/antifungal agents, retinoids, keratolytics, and surgical debridement^37,38^. We attempted to alleviate epidermal pathology using mechanistic insights derived from biophysical studies of the causative mutations^7,9,26^. We found that FFA inhibited mutant connexin hemichannels in vitro, and ameliorated the skin pathology associated with KID syndrome in a mouse model in vivo. It should be noted that obtaining direct evidence of hemichannel inhibition in the intact epidermis of a living mouse is extremely difficult and has not been done in this study. Nevertheless, these results suggested that hemichannel inhibition could provide a target to develop new therapeutic interventions^26,39,40^. They also supported the view that dysregulated hemichannel activity drives skin disease in KID syndrome.

These ideas were reinforced by the recent observation that administration of a monoclonal antibody targeting connexin hemichannels to transgenic mice expressing a mutation causing Clouston syndrome reduced skin-related pathology^41^. The antibody blocked hemichannels in vitro, including those formed by mutated human connexins^42^. Antibody treatment curtailed epidermal hyperproliferation and alleviated hypertrophic sebaceous glands in a mouse model expressing the Cx30-A88V Clouston syndrome mutation, which displayed hemichananel activity^43,44^. This supported our results with FFA in a mouse model of KID syndrome, and could be applicable to other skin diseases. In addition to KID and Clouston syndromes, aberrant hemichannel activity has been linked to palmoplantar keratoderma and deafness, palmoplantar keratoderma and congenital alopecia-1, and erythrokeratodermia variabilis et progressive^45–47^.

Topical administration of FFA stopped progression of KID epidermal pathology, but did not eliminate it. While the reasons for this are unknown, it could be related to the impact of hemichannel activity on the epidermal barrier. In KID patients, an impaired barrier contributes to susceptibility to viral, bacterial, and fungal infection^36^, and could result from increased activity of connexin hemichannels^48,49^. The epidermis maintains a Ca^2+^gradient, and Ca^2+^ is a regulator of keratinocyte differentiation^50^. KID hemichannels would disrupt this gradient, and potentially affect barrier formation^51–53^. This view is supported by mouse models of KID syndrome, where the epidermal calcium concentration was elevated in the cornified layer. The disturbed Ca^2+^ gradient was associated with an altered lipid composition in the stratum corneum, which resulted in a defective barrier^54^. We speculate that as KID lesions develop in our inducible model, the barrier degrades which allows topically applied FFA to easily penetrate into the epidermis. As the FFA inhibits mutant Cx26 activity, hemichannel Ca^2+^ flux is reduced, and the barrier recovers. Barrier recovery may reduce further FFA penetration into the epidermis, where inducible transgenic expression of mutant hemichannels would be ongoing. An equilibrium could result that would maintain the milder keratoderma seen in the treated animals, while simultaneously preventing elimination of the KID pathology in the epidermis. This is a limitation of the mouse model, where mutant connexin expression is constantly high, in contrast to human epidermis where Cx26 expression would be expected to decline as pathology improved^4,55,56^.

Decades of research have led to the point where new treatments can now be based on mechanistic data. It has been established that connexin mutations augment hemichannel activity, and contribute to epidermal pathology through altered Ca^2+^ flux^49,51,54,57^. Mouse models replicate human epidermal pathology^13,43,54,58^, and strategies to disrupt hemichannel activity have been developed^40,41,59–62^. As hemichannel dysfunction may contribute to pathological mechanisms in other connexinopathies^21,40,60,61^, our finding that topical FFA treatment ameliorated the epidermal pathology of KID syndrome could have impact across additional hemichannel-dependent human disorders.

## Methods

### Oocyte microinjection

Human Cx26-G45E in pCS2^+^^8^ was linearized with Not1 and mRNA was transcribed (SP6 mMessage mMachine, Ambion, Austin, TX). *Xenopus laevis* oocytes were purchased (Xenopus 1, Dexter, MI) and cultured in modified Barth’s (MB) medium^10^. Oocytes were injected with 10 ng of antisense oligonucleotide against *Xenopus* Cx38^63^, followed by Cx26-G45E mRNA (5 ng/cell). Antisense Cx38 oligonucleotide treated oocytes injected with water, instead of mRNA, served as controls.

### Hemichannel recording

Hemichannel currents were recorded 24 h after mRNA injection using a GeneClamp 500 amplifier, Digidata 1440A, and pClamp software (Axon, Foster City, CA). Electrodes (World Precision Instruments, Sarasota, FL) were pulled to 1–2 MΩ resistance (Narishige, Tokyo, Japan) and filled with 3 M KCl, 10 mM EGTA, and 10 mM HEPES, pH 7.4. Cells were recorded in MB medium without calcium^8^. Current–voltage curves were obtained by clamping cells at − 40 mV and imposing voltage steps in 10 mV increments from − 30 to + 60 mV^46^.

### Drug testing

Extracellular solutions were exchanged using gravity perfusion^26^. FFA (MilliporeSigma, Burlington, MA) was dissolved in DMSO at a concentration of 200 mM, and serially diluted into MB medium without calcium at concentrations between 1 and 500 µM. I–V curves were obtained before and after exchange of the extracellular medium with a known concentration of FFA. Percent inhibition was plotted against drug concentration and fit to a sigmoidal function (OriginLab, Northampton, MA) to determine the IC_50_.

### Animals

Animal work was approved by the Stony Brook University IACUC, and conducted according to the NIH Guide for the Care and Use of Laboratory Animals and in compliance with the ARRIVE guidelines. Male and female Cx26-G45E transgenic mice in the SKH1 outbred and uncharacterized genetic background were used^13,32^, no sex based differences were observed^13^. When fed a doxycycline containing diet (625 mg/kg, Envigo, Indianapolis, IN), these mice express Cx26-G45E and enhanced green fluorescent protein (EGFP) as independent proteins in epidermal keratinocytes. Animals were genotyped by PCR amplification of tail genomic DNA (Choice Taq, Thompson Scientific, Swedesboro, NJ) as described^13^.

### In-vivo fluorescent imaging

EGFP fluorescence was monitored in transgenic animals using an In Vivo Imaging System (Lumina III, Perkin Elmer, Melville, NY). Mice were anesthetized with isoflurane and whole body dorsal fluorescent images were acquired. EGFP fluorescence was recorded as the total radiant efficiency (p/sec/cm^2^/sr/μW/cm^2^) for each animal subject.

### Drug treatment

Transgenic mice were initially induced with doxycycline for 3–5 days and subjected to IVIS imaging to select cohorts with similar levels of disease progression. Mice were photographed with a digital camera (Canon, Melville, NY) and then treated topically with FFA, or vehicle, while still under isoflurane anesthesia following IVIS imaging. Mobilisin cream (Crinos, Milan, Italy) containing 3% FFA (~ 110 mM) was purchased from an Italian pharmacy (Farmacia Etzi-Delitala, Olbia, Italy). FFA (MilliporeSigma, Burlington, MA) was dissolved in DMSO at 200 mM, and mixed with Eucerin Cream (Beiersdorf, Wilton, CT) at a 1:5.7 ratio (30 mM final FFA). For vehicle controls, DMSO was mixed with Eucerin Cream at the same 1:5.7 ratio. Mobilisin, FFA/Eucerin, or DMSO/Eucerin (~ 0.5 ml/ mouse) were applied to the dorsal epidermis. Mice were maintained under isoflurane anesthesia for 10 min in a warmed chamber to allow absorption of the cream. Statistical significance was determined using one way ANOVA, or the 2-tailed t-test (OriginLab Corp).

### Histology

Skin was fixed in 4% formaldehyde in PBS for 24 h at room temperature. Tissues were rinsed with PBS, dehydrated through an ethanol series, and embedded in paraffin. 2–3 µm sections were cut, deparaffinized, and stained with hematoxylin–eosin^13^.

### Immunocytochemistry

Skin was fixed in a 1% formaldehyde in PBS for 1 h at room temperature. Tissues were rinsed with PBS, immersed in Optimal Cutting Temperature compound (Ted Pella, Redding, CA), and frozen. 8–10 μm sections were cut on a cryotome, dried onto glass slides, and stained with polyclonal rabbit antibodies against Cx26 (Zymed, San Francisco, CA), washed with 0.1% Triton X-100/PBS, incubated with Cy3-conjugated anti-rabbit secondary antibodies (Jackson ImmunoResearch, West Grove, PA), washed with 0.1% Triton X-100/PBS, and mounted using Vectashield with 4′,6-diamidino-2-phenylindole (Vector, Burlingame, CA).
